# Maternal obesity causes fetal hypothalamic insulin resistance and disrupts development of hypothalamic feeding pathways

**DOI:** 10.1016/j.molmet.2020.101079

**Published:** 2020-09-09

**Authors:** L Dearden, S Buller, IC Furigo, D.S. Fernandez-Twinn, SE Ozanne

**Affiliations:** 1 University of Cambridge Metabolic Research Laboratories, Institute of Metabolic Science, Level 4, Box 289, Addenbrooke's Hospital, Cambridge, CB20QQ, United Kingdom

**Keywords:** Maternal obesity, Hypothalamus, Insulin, Neurogenesis, Notch

## Abstract

**Objective:**

Perinatal exposure to maternal obesity results in predisposition of offspring to develop obesity later in life. Increased weight gain in offspring exposed to maternal obesity is usually associated with hyperphagia, implicating altered central regulation of food intake as a cause. We aimed to define how maternal obesity impacts early development of the hypothalamus to program lasting dysfunction in feeding regulatory pathways.

**Methods:**

Mice offspring of diet-induced obese mothers were compared to the offspring of lean control mothers*.* We analysed gene expression in the fetal hypothalamus, alongside neurosphere assays to investigate the effects of maternal obesity on neural progenitor cell proliferation *in vitro*. Western blotting was used to investigate the insulin signalling pathway in the fetal hypothalamus. Characterisation of cell type and neuropeptide profile in adulthood was linked with analyses of feeding behaviour.

**Results:**

There was a reduction in the expression of proliferative genes in the fetal hypothalamus of offspring exposed to maternal obesity. This reduction in proliferation was maintained *in vitro* when hypothalamic neural progenitor cells were grown as neurospheres. Hypothalamic fetal gene expression and neurosphere growth correlated with maternal body weight and insulin levels. Foetuses of obese mothers showed hypothalamic insulin resistance, which may be causative of reduced proliferation. Furthermore, maternal obesity activated the Notch signalling pathway in neonatal offspring hypothalamus, resulting in decreased neurogenesis. Adult offspring of obese mothers displayed an altered ratio of anorexigenic and orexigenic signals in the arcuate nucleus, associated with an inability to maintain energy homeostasis when metabolically challenged.

**Conclusions:**

These findings show that maternal obesity alters the molecular signature in the developing hypothalamus, which is associated with disrupted growth and development of hypothalamic precursor cells and defective feeding regulation in adulthood. This is the first report of fetal hypothalamic insulin resistance in an obese pregnancy and suggests a mechanism by which maternal obesity causes permanent changes to hypothalamic structure and function.

## Highlights

• Exposure to maternal obesity reduces hypothalamic neural progenitor cell growth.
• Maternal obesity activates hypothalamic Notch signalling and reduces neurogenesis.
• Maternal obesity causes fetal hypothalamic insulin resistance.
• Maternal obesity alters the ratio of anorexigenic/orexigenic signals in ARC.
• Changes in food intake precede increased adiposity in offspring of obese dams.

## 1 Introduction

The number of pregnancies complicated by obesity is increasing in line with the worldwide obesity crisis. Recent figures show that more than 50% of pregnancies in the UK are in overweight or obese women [1]. Obesity is associated with complications for both mother and baby during pregnancy and birth, including increased still-birth, caesarean rate and shoulder dystopia. In addition to these immediate complications, studies have shown that the children of obese mothers are more likely to develop obesity, type 2 diabetes and cardiovascular disease later in life [2]. Increasing evidence suggests that this does not simply reflect transmission of obesogenic genes from mother to child. For example, studies of siblings discordant for *in utero* exposure to maternal obesity show the sibling exposed to maternal obesity has increased adiposity and greater insulin resistance [3], demonstrating that the increased risk of metabolic disease is in addition to heritable or current lifestyle factors. Animal studies have shown causal relationships between obesity during pregnancy and increased weight gain of the offspring. They further demonstrated that this increased weight gain is often preceded by hyperphagia, implicating altered neuronal control of food intake as an underlying mechanism [4]. The hypothalamus plays a critical role within the brain in maintaining energy homeostasis by sensing and responding to changes in nutrient status, and it has recently been demonstrated that rare genetic variants affecting early hypothalamic development are associated with obesity [5]. Although several studies have shown alterations in offspring hypothalamic anatomy or gene expression in adulthood after exposure to maternal obesity, the fetal origins of these alterations remain unclear.

Hypothalamic development consists of sequential cellular processes that can be categorised into three major categories: 1) proliferation of neural progenitor cells (NPCs) and birth of new neurons, 2) the migration of newborn cells to their final destination and 3) neurite extension and formation of functional circuits. Experiments in rodents have demonstrated that the vast majority of neurons in hypothalamic nuclei known to control energy balance are generated between embryonic day (E) 12 and E16 [6], and that the majority of hypothalamic projections develop in the early postnatal period [7]. Differentiation of NPCs into mature neurons is governed by a complex interplay of molecular pathways, including the signalling molecule Notch and its downstream regulators in the Hes family, which act through a series of basic helix-loop-helix transcription factors to maintain NPCs in an immature state and thus inhibit neuronal differentiation [[8], [9], [10], [11]]. Recent studies have shown that inhibition of Notch signalling in hypothalamic nuclei is associated with decreased expression of Hes1 and a concomitant increased Mash1 expression, coincident with increased proliferation and accumulation of mature hypothalamic neurons [12,13].

Several animal models have shown that exposure to maternal obesity disrupts feeding control in adult offspring; however, the early mechanisms underlying this phenotype remain largely elusive. There is now robust evidence that in rodent models, exposure to an obesogenic environment during the perinatal period disrupts the proper formation of intra- and extra-hypothalamic projections, and this is associated with hyperphagia [[14], [15], [16]]. This may be due to the altered levels of hormones, such as leptin and ghrelin, which are required for hypothalamic development and can be altered in an obese pregnancy [17,18], or the disrupted expression of axon guidance molecules at key developmental time points [19]. The impact of an obesogenic *in utero* environment on earlier developmental processes, such as NPC proliferation and neurogenesis, is unknown. However, Plagemann et al. have demonstrated that offspring exposure to gestational diabetes results in the malformation of hypothalamic nuclei, which may be secondary to reduced neuron formation [[20], [21], [22]]. Indeed, it has been demonstrated that maternal obesity alters Notch signalling in cerebral neural stem cells [23], and decreased neurogenesis and neuronal differentiation has been reported in the hippocampus of adult offspring exposed to maternal obesity [24], which may be due to altered Notch signalling [25].

One of the few studies to show a causative link between disrupted hypothalamic development and later life functionality is the study by Vogt et al., which showed that the effects of maternal obesity on offspring hypothalamic control of glucose homeostasis are mediated via aberrant hypothalamic insulin signalling in the post-natal period [14]. Insulin is classically thought of as a growth factor and has strong survival-promoting effects in neurons. Indeed, access to recombinant insulin in the 1980s was a pivotal point in the ability to transform neuronal precursors into mature neurons in culture [26,27]. Other early studies showed that insulin has a neurotrophic function and promotes neurite outgrowth in cultured neuronal cells [[28], [29], [30]]. It has recently been reported that the effects of insulin on neuronal survival are influenced by the nutritional status of the animal in which the neurons develop [31].

Obese pregnancies are often associated with both maternal and fetal hyperinsulinaemia, and high levels of maternal insulin and glucose during pregnancy may disrupt fetal insulin signalling, as evidenced by human studies showing that offspring of obese mothers develop hallmarks of insulin resistance *in utero* [32]. While animal studies have shown that exposure to maternal obesity programs hypothalamic and hippocampal insulin resistance in offspring in adulthood [33,34], no studies to date have examined insulin signalling in fetal brains exposed to maternal obesity in order to establish how early insulin resistance occurs. Given the importance of insulin as a neuronal survival signal required for normal brain development, disrupted insulin signalling during fetal life could cause lasting impairments in hypothalamic development that result in lifelong metabolic dysregulations.

We therefore sought to establish how maternal obesity impacts the early developmental processes of neurogenesis and neuronal differentiation in the offspring hypothalamus and examine hypothalamic insulin signalling in foetuses exposed to maternal obesity. Our results show that maternal obesity disrupts early hypothalamic development, including a reduction in proliferation of NPCs, while at the same developmental timepoint altering fetal hypothalamic insulin signalling machinery. These changes to the developing hypothalamus are associated with disrupted feeding behaviour in adulthood, suggesting that fetal insulin resistance may underlie the development of long-term feeding dysregulation in offspring exposed to maternal obesity.

## 2 Methods

### 2.1 Animal model

All animal experiments were approved by the UK Home Office and the University of Cambridge Animal Welfare and Ethical Review Body. To study the effects of exposure to obesity on the offspring hypothalamus, we utilised a mouse model previously published by our group [35]. Briefly, female C57BL/6J mice were randomly assigned to either a control chow diet (RM1; 3.29 kcal/g; Special Dietary Services, UK) or a highly palatable obesogenic diet consisting of a high-fat diet (45% kCal from fat; 4.54 kcal/g; Special Dietary Services, UK), supplemented with sweetened condensed milk (3.26 kcal/g; Nestle, UK) fortified with mineral and vitamin mix AIN93G. Consistent with our previous studies [36] and as expected, dams fed the obesogenic diet consumed more calories throughout pregnancy than those fed the control diet (average kcal/day: control 16.26 ± 0.78 vs obese 35.62 ± 3.23; p < 0.001). Once females from the obesogenic diet group had achieved a body composition around 30–35% fat and body weight exceeding 35 g, they were mated for pregnancy along with age-matched control dams (10–12 weeks of age). Dams remained on their respective diets throughout pregnancy and the post-natal period. A subset of dams was culled on E13 to collect the fetal hypothalamus. Maternal body weight and for some dams maternal serum was collected on day of dissection for later analysis. Remaining dams were allowed to litter naturally, and neonatal hypothalamic tissue was collected from pups on post-natal day 2 (PND2) or offspring remained with dam until weaning at 3 weeks of age. Litters were standardised to 6 pups on PND2 (favouring males) to ensure standardised nutrition. For each timepoint (E13, PND2, 8 weeks), one animal from each litter was included in each separate experiment; therefore, the statistical number comes from the dam.

### 2.2 Hypothalamic tissue collection

*E13*: Dams were culled on day 13 of pregnancy (day of plug = day 0), and brains were swiftly collected from foetuses of both sexes. The hypothalamus was then carefully dissected from the rest of the brain under a stereomicroscope. *PND2*: Neonates of both sexes were sacrificed by cervical dislocation on PND2 (day of birth = day 0), and the whole hypothalamus was rapidly dissected. *Eight weeks*: Male mice were culled by cervical dislocation, and brains were immediately removed and stored at −80 °C until sectioning at 12 μm on a cryostat (Leica Biosystems). PVH and ARC tissue was collected from brain sections using laser capture microdissection with a PALM Robot Microbean (Carl-Zeiss). All hypothalamic tissues were stored in QIAzol lysis buffer at −20 °C until RNA extraction.

### 2.3 Food intake and body composition analyses of adult offspring

At weaning, male mice from control or obese mothers were randomly assigned to either a control (chow, RM1 as above) or obesogenic diet (HFD, as above), creating 4 experimental offspring groups for adult studies: offspring born to control mothers weaned onto a chow diet (Off-C chow), offspring born to control mothers weaned onto an HFD (Off-C HFD), offspring born to obese mothers weaned onto a chow diet (Off-Ob chow), offspring born to obese mothers weaned onto an HFD (Off-Ob HFD). *Ad libitum* food intake was measured over a 24-hour period at 6 weeks and 8 weeks of age. For fasting/refeeding experiments, offspring were fasted overnight (1600–0800) at 6 weeks of age. A defined amount of food was returned in plastic dishes into the home cage, and food intake was measured after 1, 2, 3 and 6 h. Adiposity was measured in fed offspring at 6 and 8 weeks of age using a TDNMR machine (Bruker).

### 2.4 Insulin enzyme-linked immunosorbent assay (ELISA)

Blood samples from dams were collected by cardiac puncture and centrifuged for serum collection. An ultra-sensitive mouse insulin ELISA (Crystal Chem Inc, Illinois, USA) was used to determine plasma levels of insulin.

### 2.5 Hypothalamic neurosphere culture

The fetal hypothalamus was collected on E13 as described above. Following dissection, tissue was mechanically dissociated and centrifuged to remove debris. Supernatant containing NPCs was cultured in Neurocult™ proliferation media (Stem Cell Technologies, Cambridge, UK) containing recombinant human EGF (Stem Cell Technologies, Cambridge, UK) according to the manufacturers’ instructions. Cells were cultured for 8 days *in vitro* (DIV). On each day, a researcher blinded to the treatment groups took 20 images using a grid system throughout the flask. ImageJ was used to quantify the number and size of neurospheres in the images and an average for each flask per day was generated. On day 8, neurospheres floating in media were centrifuged at 100 G for 5 min to pellet neurospheres, and pelleted neurospheres were dissociated to create a single cell suspension. Cell number was counted using a Neubauer chamber.

### 2.6 RNA extraction and gene expression analysis

All RNA extraction from tissue and cultured cells was performed using the RNeasy plus micro Kit (Qiagen), following manufacturers’ protocols. cDNA was reverse transcribed using a RevertAid First Strand cDNA Synthesis Kit (Thermo Scientific). Gene expression was assessed using SYBR Green or Taqman technologies and a StepOnePlus Real-Time PCR System. Relative quantification of mRNA was calculated by the 2-ΔΔCt method. Data were normalised to the housekeeping gene *Sdha* (expression of which did not change between groups) and values were reported as fold changes compared to values obtained from the control group (set at 1.0). Primers for SYBR Green-based quantitative polymerase chain reaction (qPCR) were obtained from Sigma Aldrich; primer sequences are listed in Table 1. TaqMan probes were obtained from ThermoFisher Scientific and are listed in Table 2.

**Table 1:** Sequences of primers (purchased from Sigma Aldrich) used in SYBR green quantitative real-time PCR analysis.

Gene name	Forward primer sequence (5′-3′)	Reverse primer sequence (5′-3′)
*Bub1b*	GCA GAA AGC GGC ATT TGA ATC TG	GTC CTT CTA TCG CTC TCT CCA C
*c-myc*	TGA CCT AAC TCG AGG AGG AGC TGG ATT C	AAG TTT GAG GCA GTT AAA ATT ATG GCT GAA GC
*Gfap*	CCA GCT TCG AGC CAA GGA	GAA GCT CCG CTT GGT AGA CA
*Ki67*	CCT TTG CTG TCC CCG AAG A	GGC TTC TCA TCT GTT GCT TCC T
*NeuN*	GGC AAT GGT GGG ACT CAA AA	GGG ACC CGC TCC TTC AAC
*Ngn2*	CTG GAG CCG CGT AGG ATG T	CAG CAT CAG TAC CTC CTC TTC C
*Pcna*	TGC TCT GAG GTA CCT GAA CT	TGC TTC CTC ATC TTC AAT CT
*Sdha*	TCG ACA GGG GAA TGG TTT TGG	TAA TCT TC CTG GCA TGG GC

**Table 2:** Assay ID for probes (purchased from ThermoFisher) used in Taqman quantitative real-time PCR analysis.

Gene name	Taqman assay ID
*Ascl1*	Mm03058063_m1
*Hes1*	Mm01342805_m1
*Hes5*	Mm00439311_g1
*Notch1*	Mm00627185_m1
*Sdha*	Mm01352366_m1

### 2.7 Protein extraction, sodium dodecyl sulphate polyacrylamide gel electrophoresis (SDS PAGE) and western blotting

The fetal hypothalamus was dissected as described above. Protein was extracted in lysis buffer (50 mmol/l HEPES [pH 8], 150 mmol/l NaCl, 1% (wt/vol.) Triton X-100, 1 mmol/l Na_3_VO_4_, 30 mmol/l NaFl, 10 mmol/l Na_4_P_2_O_7_, 10 mmol/l EDTA (all Sigma–Aldrich) and a 1:200 dilution of protease inhibitor cocktail set III [Merck-Millipore, Watford, UK]). Total protein concentration of lysates was determined using a bicinchoninic acid kit (Sigma–Aldrich) and samples diluted in Laemmli buffer. Samples (5 μg protein) were loaded onto 8% polyacrylamide gels for electrophoresis and transferred to a polyvinylidene difluoride (PVDF) membrane (Merck-Millipore). The membrane was rinsed in Tris-buffered saline with Tween (TBST) and then blocked in 5% (wt/vol.) bovine serum albumin (BSA) in TBST for 1 h, followed by overnight incubation with the primary antibodies listed in Table 3. Following washes in TBST, membranes were incubated for 1 h in horseradish-peroxidase linked secondary antibodies (Table 3) diluted 1:20,000 in 5% BSA in TBST. Membranes were washed in TBST before detection using chemiluminescence substrate (Immobilon Forte, Millipore) on the ChemiDoc Imaging system (Bio-Rad, Hemel Hempstead, UK). Protein abundance was quantified by band densitometry using Image Lab 5.1 software (Bio-Rad) and normalised to total transferred protein as visualised and quantified following Coomasie-250 staining of the membranes after western blotting.

**Table 3:** Supplier details and dilutions for primary and secondary antibodies used in Western blotting experiments.

Antibody name	Supplier	Dilution
INSR	Santa Cruz (SC-711)	1:200
IRS1	Millipore (06–248)	1:1,000
P- IRS1 (SER 307)	Upstate Biotech (07–247)	1:1,000
AKT	Cell Signalling (2967)	1:1,000
P- AKT (THR 308)	Cell Signalling (9275)	1:1,000
ERK1/2	Cell Signalling (9102)	1:1,000
P- ERK1/2	Cell Signalling (9101)	1:1,000
Anti-rabbit-IgG-HRP	Abcam (AB6721)	1:20,000
Anti-mouse-IgG-HRP	Abcam (AB6728)	1:20,000

### 2.8 Immunohistochemical analysis

For immunostaining of adult tissue, mice were terminally anaesthetised and transcardially perfused with 4% paraformaldehyde. Tissue sections were processed for immunofluorescence using standard procedures. The antibodies used for immunohistochemistry were: POMC (H029-30; Phoenix Pharmaceuticals) and donkey anti-rabbit Alexa Fluor Plus 488 (Thermo Fisher). Images were acquired using a Zeiss LSM 510 confocal system equipped with a 10X objective from Bregma −1.35 mm to −1.65 mm. Slides were numerically coded to obscure the treatment group. Quantitative analysis of cell number was performed using ImageJ analysis software (National Institutes of Health, NIH). Briefly, images were binarised to isolate labelled particles from the background and to compensate for differences in fluorescence intensity. Then, watershed segmentation was used to define individual cells and cell number automatically counted. For a random set of samples, POMC-positive cells were manually counted to ensure automated analysis was an accurate representation of images.

### 2.9 Statistical analyses

Data were analysed using Prism 8 (GraphPad, La Jolla, USA) by different tests according to the numbers of groups: unpaired t-test for experiments with two treatment groups (with Welch's correction where appropriate), or two-way analysis of variance (ANOVA) with multiple comparisons test for experiments with 2 more groups over a time course. Pearson correlations were computed to determine correlation between two factors. Outliers were detected by Grubb's method and excluded as indicated in figure legends. The data are shown as mean ± SEM.

## 3 Results

### 3.1 Maternal obesity reduces proliferation of offspring hypothalamic neural progenitor cells

**Figure 1:**
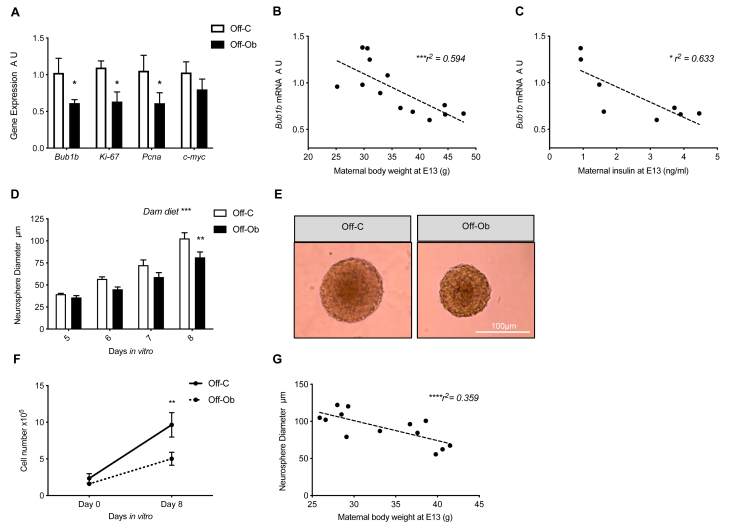
Maternal obesity reduces proliferation of hypothalamic neural progenitor cells. A) Quantitative real-time PCR analysis of *Bub1b*, *Ki67*, *Pcna* and *Cmyc* in fetal hypothalamic tissue (A.U., expressed as relative to Off-C, n = 6–7) B) Correlation between maternal body weight (g) at E13 and fetal hypothalamic expression of *Bub1b* (n = 13) C) Correlation between maternal serum insulin (ng/ml) at E13 and fetal hypothalamic expression of *Bub1b* (n = 8) D) Diameter of neurospheres grown from fetal hypothalamic NPC after 5–8 DIV (significant effect of dam diet over time course by two-way ANOVA; n = 7–8) E) Representative images of neurospheres grown from control and obese fetal hypothalamus after 8 DIV (scale bar = 100 μm) F) Dissociated cell number at the beginning of neurosphere culture (0DIV) and after 8DIV (n = 7) G) Correlation between maternal body weight (g) at E13 and neurosphere width after 8 DIV (n = 13). Off-C = white bars or solid line, Off-Ob = black bars or dashed line. Data are presented as mean +/− SEM. ∗p < 0.05, ∗∗p < 0.01, ∗∗∗p < 0.001. Outlier was excluded from (A) *Pcna*; Off-C (Grubb's method, alpha = 0.2, G = 2.198, outlier excluded value 4.42).

Analysis of E13 fetal hypothalamic tissue showed a significant decrease in the expression of proliferative gene markers *Bub1b*, *Ki67* and *Pcna* in the foetuses of an obese pregnancy (Figure 1A; all p < 0.05). Reduction in gene expression for all three genes was significantly correlated with maternal body weight as assessed by Pearson's correlation (*Bub1b* p < 0.01, r^2^ = 0.594; *Ki67* p < 0.05, r^2^ = 0.435; *Pcna* p < 0.05, r^2^ = 0.435) and *Bub1b* and *Ki67* also correlated with maternal serum insulin concentration on day 13 of pregnancy (Bub1b p < 0.01, r^2^ = 0.633; Ki67 p < 0.05, r^2^ = 0.489). Example graphs are shown for *Bub1b,* demonstrating the negative correlation between fetal mRNA expression and maternal body weight (Fig 1B) and maternal serum insulin concentration (Fig 1C).

To examine the programmed effects of exposure to maternal obesity during pregnancy on the proliferative potential of hypothalamic NPCs outside of the *in utero* environment, we utilised a proliferative neurosphere assay in which culture conditions can be strictly controlled. The fetal hypothalamus was extracted from the brains of foetuses from both control and obese pregnancies on E13 and NPCs grown in identical culture conditions to form neurospheres. Neurospheres began to form from NPCs after 3 DIV. There was no difference in the number of neurospheres that formed *in vitro* from control or obese pregnancies after 3 DIV (average per field of view: Control 2.83 ± 0.70 SEM vs Obese 2.44 ± 0.53 SEM). Neurospheres generated from hypothalamic NPCs from an obese pregnancy were significantly smaller after 8 DIV than those from a control pregnancy (Figure 1D,E; p < 0.01 neurospheres from Off-Ob significantly smaller than Off-C after 8 DIV, p < 0.001 significant effect of maternal diet over time course). After 8 DIV, neurospheres were chemically and mechanically dissociated into a single cell suspension to analyse the number of cells contained within the neurosphere. Neurospheres generated from hypothalamic NPCs from an obese pregnancy contained significantly fewer cells than those from a control pregnancy (Figure 1F; p < 0.01), suggesting that the decreased diameter of these neurospheres was due to them containing fewer cells rather than smaller cells. The size of neurospheres after 8 DIV was negatively correlated to maternal body weight at E13 across the full range of body weight; the heavier the mother was on the day of dissection, the less was the growth shown by the neurospheres generated from fetal hypothalamic NPCs in culture (Figure 1G; p < 0.001 r^2^ = 0.359).

### 3.2 Maternal obesity disrupts fetal hypothalamic insulin signalling

**Figure 2:**
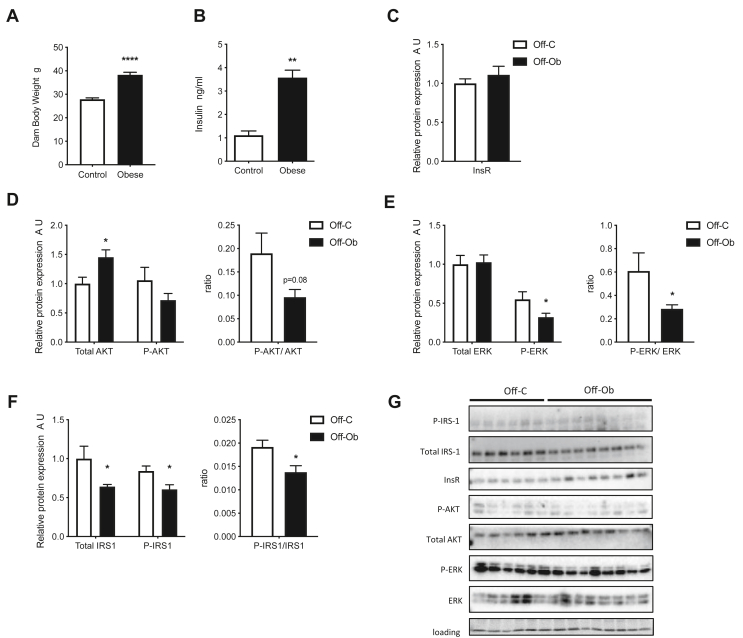
Maternal obesity impacts on fetal hypothalamic insulin signalling pathways. A) Maternal body weight (g) and B) fed serum insulin levels (ng/ml) on embryonic day 13 (n = 4–7) C) Expression of InsR protein in fetal hypothalamus (A.U., expressed as relative to Off-C; n = 6–8) D-F) Expression of total protein and phosphorylated protein (A.U., expressed as relative to Off-C) and the ratio of phosphorylated/total protein for AKT, ERK and IRS1 (n = 6–8) G) Images of western blots represented by graphs in Figure 2 plus loading control. A-B) Control dam = white bars, Obese dam = black bars. C–F) Off-C = white bars, Off-Ob = black bars. b chow = black bars, Off-C HFD = white patterned bars, Off-Ob HFD = black patterned bars. Data are presented as mean +/− SEM. ∗p < 0.05, ∗∗p < 0.01, ∗∗∗∗p < 0.0001. Outlier was excluded from (B) control (Grubb's method, alpha = 0.2, G = 1.493, outlier excluded value = 6.621).

Obese mothers displayed a significant increase in both body weight (Figure 2A; p < 0.001) and serum insulin levels (Figure 2B; p < 0.05) compared to control mothers by day 13 of pregnancy due to consumption of the obesogenic diet. To ascertain what effect exposure to high maternal adiposity and insulin has on fetal hypothalamic insulin sensing machinery, the expression and phosphorylation of key components of the insulin signalling pathway was examined at both the mRNA and protein level in the E13 fetal hypothalamus. There were no changes in the mRNA levels of the insulin receptor (*InsR*) (OffC 1.00 ± 0.04 SEM vs OffOb 0.96 ± 0.03 SEM), *Akt* (OffC 1.03 ± 0.02 SEM vs OffOb 0.95 ± 0.03 SEM) or insulin receptor substrate-1 (*Irs1*) (OffC 1.04 ± 0.02 SEM vs OffOb 1.01 ± 0.02 SEM) between the fetal hypothalamus from a control or obese pregnancy. There was also no difference in the protein expression of the InsR in a fetal hypothalamus from an obese pregnancy (Fig 2C). However, a significant increase was found in fetal hypothalamic total AKT protein levels in an obese pregnancy (Figure 2D; p < 0.01), but no change was found in phosphorylated AKT (P-AKT) or the ratio between P-AKT to total AKT (Fig 2D). There was no change in total ERK protein levels between control and obese pregnancies, but a decrease in phosphorylated ERK (P-ERK) and a decrease in the ratio of P-ERK to total ERK (Figure 2E; p < 0.05). There was also a decrease in the fetal hypothalamic levels of total IRS1, phosphorylated IRS1 (P-IRS1) and the ratio of P-IRS1 to total IRS1 (Figure 2F; p < 0.05). Original scans of western blots of all proteins are shown in Figure 2G.

### 3.3 Maternal obesity modulates expression of notch signalling pathway in neonatal offspring hypothalamus

**Figure 3:**
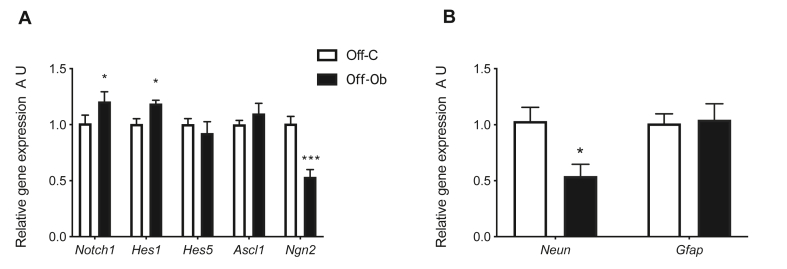
Exposure to maternal obesity activates the hypothalamic Notch signalling pathway and reduces neuronal markers. A) Quantitative real-time PCR analysis of *Notch1*, *Hes1*, *Hes5, Ascl1* and *Ngn2* in neonatal hypothalamic tissue (A.U., expressed as relative to Off-C, n = 5–6) B) Quantitative real-time PCR analysis of *NeuN* and *Gfap* in the neonatal hypothalamic tissue (A.U., expressed as relative to Off-C, n = 5–6). Off-C = white bars, Off-Ob = black bars. Data are presented as mean +/− SEM. ∗p < 0.05,∗∗∗p < 0.001.

Gene expression of key components of the Notch signalling pathway was quantified in neonatal (PND2) hypothalamus of offspring of control or obese mothers. We observed a significant increase in the expression of genes encoding *Notch1* and *Hes1* in the hypothalamus of offspring of obese mothers (Figure 3A; both p < 0.05). There was no difference in the hypothalamic expression of *Hes5*, *Ascl1* or *Ngn1* between offspring of control or obese mothers. There was a significant decrease in the expression of *Ngn2* in the hypothalamus of offspring exposed to maternal obesity at PND2 (Figure 3A; p < 0.001). These gene expression changes are suggestive of inhibition of pro-neural differentiation in the hypothalamus of offspring exposed to maternal obesity. We therefore examined the expression of classical cell type markers for neurons (Neun) and astrocytes (Gfap) in the same animals. We observed a significant decrease in the gene expression of the neuronal marker *NeuN* in the hypothalamus of offspring from obese mothers compared to controls at PND2 (Figure 3B; p < 0.05).

### 3.4 Maternal obesity alters the hypothalamic neuropeptide profile and feeding behaviour in adult offspring

**Figure 4:**
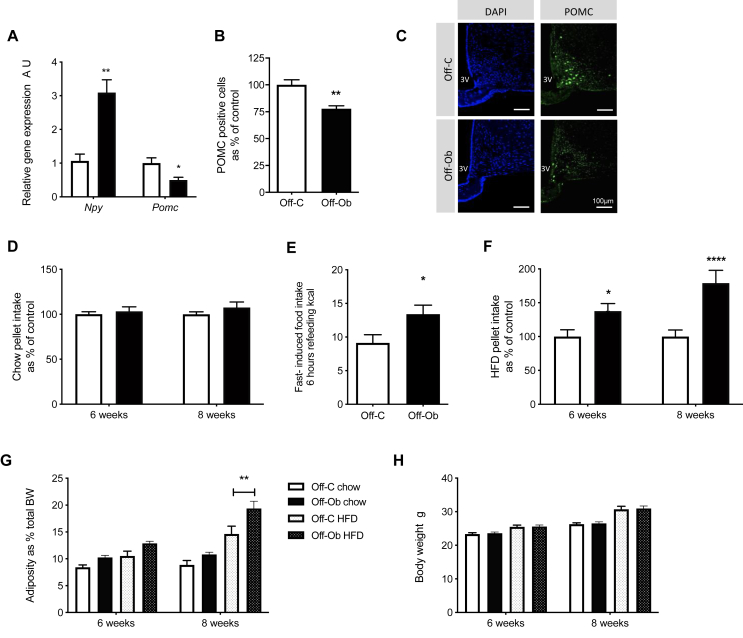
Altered hypothalamic development is associated with disrupted feeding control and increased adiposity in offspring of obese mothers in adulthood. A) Quantitative real-time PCR analysis of *Npy* and *Pomc* in arcuate nucleus of 8-week-old mice (A.U., expressed as relative to Off-C, n = 5–6) B) Number of POMC-positive cells in arcuate nucleus of 8-week-old mice as asssessed by immunofluorescence (A.U., expressed as relative to Off-C, n = 5–6) C) Representative images of POMC staining in the ARC of 8 week old offspring of control and obese mothers (scale bar = 100 μm) D) Ad lib intake of chow pellet in offspring at 6 and 8 weeks of age (A.U., expressed as relative to Off-C, n = 15) E) 6-h cumulative food intake of chow pellet after overnight fast (kCal, n = 7–10) F) Ad lib intake of HFD pellet in offspring at 6 and 8 weeks of age (A.U., expressed as relative to Off-C, n = 20) G) Adipose tissue mass as percentage of total body weight in offspring at 6 and 8 weeks of age (n = 13, 12, 20, 13) H) Body weight in offspring at 6 and 8 weeks of age (n = 14, 13, 21, 15). A-F) Off-C = white bars, Off-Ob = black bars. G&H) Off-C chow = white bars, Off-Ob chow = black bars, Off-C HFD = white patterned bars, Off-Ob HFD = black patterned bars. Data are presented as mean +/− SEM. ∗p < 0.05,∗∗p < 0.01, ∗∗∗∗p < 0.0001. Outlier was excluded from (A) *Npy*; Off-Ob (Grubb's method, alpha = 0.2, G = 1.739, outlier excluded value 0.08), and (E) Off-C (Grubb's method, alpha = 0.2, G = 2.512, outlier excluded value = 29.6).

To examine the effect of the changes during early hypothalamic development on hypothalamic neuropeptide profile in adult animals, we examined the expression of canonical feeding-related neuropeptide genes pro-opiomelanocortin (POMC) and neuropeptide Y (NPY) in the ARC of 8-week-old offspring from control and obese mothers weaned onto a chow diet. Offspring of obese mothers showed a significant reduction in mRNA expression of the anorectic neuropeptide *Pomc* and a reduction in the number of POMC positive neurons as assessed by immunofluorescence at 8 weeks of age (Figure 4A; p < 0.05 & 4B; p < 0.01, representative images Fig 4C). We also observed a significant increase in the gene expression of orexigenic *Npy* (Figure 4A; p < 0.01).

To assess the effects of altered hypothalamic neuropeptide profile on the ability of offspring to maintain energy balance, we examined *ad lib* food intake of a chow diet, as well as responses to two differing metabolic challenges (consumption of an obesogenic diet and response to fasting). There was no difference in *ad lib* intake of a chow diet between offspring of control or obese mothers at 6 or 8 weeks of age (Fig 4D). Despite no change in *ad lib* intake of chow diet, the offspring of obese mothers fed a chow diet show increased rebound feeding following a fast (Figure 4E; p < 0.05). The offspring of obese mothers also showed increased *ad lib* food intake at both 6 and 8 weeks of age when weaned onto an HFD compared to their control counterparts (Figure 4F; 6 weeks p < 0.05, 8 weeks p < 0.0001). These changes in food intake precede changes in adiposity in the animals. There was no difference in adiposity (as a percentage of total body weight) between offspring of control or obese mothers, regardless of the diet they are weaned onto, at 6 weeks of age (Fig 4G). However, at 8 weeks of age, the offspring of obese mothers show increased percentage of adiposity compared to the offspring of control mothers weaned onto the obesogenic diet (Figure 4G; p < 0.01). Total body weight (g) was increased by offspring diet, but not maternal diet (Fig 4H).

## 4 Discussion

Here, we report that exposure to maternal obesity causes reduced proliferation of hypothalamic NPCs, fetal hypothalamic insulin resistance and neonatal alterations in the Notch signalling pathway. These changes in early hypothalamic development are associated with altered hypothalamic neuropeptide profile and dysregulation of feeding control in adult offspring of obese mothers.

Impaired peripheral and central insulin sensitivity in adult offspring of obese mothers has been reported by us and others [33,34,37]. However, the current findings provide the first evidence of changes in the hypothalamic insulin signalling pathway before birth. These changes are therefore a direct result of the obese *in utero* environment, rather than a result of changes in adiposity or circulating hormone levels in adult animals. The fetal timepoints included in our study provide some of the first conclusive evidence that changes in hypothalamic development *in utero* precede changes in feeding behaviour and adiposity. Hypothalamic insulin resistance has been proposed as a contributing feature of obesity in adulthood [38], but our results suggest it can be established *in utero* and therefore could be an underlying cause in the development of disrupted energy homeostasis in individuals exposed to maternal obesity. Changes in the fetal hypothalamus present from before birth help to explain the observation that offspring exposed to maternal obesity show altered feeding control even before the onset of independent feeding, as indicated by increased milk intake in the pre-weaning period [39]. The changes we observed in protein levels of insulin signalling pathway proteins were not accompanied by changes in the corresponding mRNA levels, suggesting the mechanistic basis for altered expression is post-transcriptional, as we have previously reported in adult human and rodent adipose tissue [36,40]. The changes we have reported in fetal hypothalamic insulin signalling are during a basal, non-insulin stimulated state in the animals. The lack of insulin-stimulated data is a limitation of our study. However, it is challenging to alter fetal insulin levels non-invasively in pregnancy. Furthermore, it is not possible to manipulate fetal insulin in isolation *in vivo* as this will subsequently cause fetal hypoglycaemia, which is not representative of the hyperglycaemic/hyperinsulinaemic environment of an obese pregnancy.

It has previously been shown that neurogenesis in the ARC is reduced in adult mice with diet-induced obesity [41] and in neonatal offspring of obese mothers [42]. However, whether exposure to an obesogenic environment *in utero* impacts proliferation in the fetal hypothalamus during development was unknown. The current study shows reduced proliferative markers in the fetal hypothalamus of an obese pregnancy. These changes may be stage of development specific, as two recent papers have demonstrated increased proliferation in the hypothalamus of offspring of obese mothers in young adulthood [42,43]. The cause of the reduced proliferation we observed in the fetal hypothalamus remains unclear. Our results show that the expression of proliferative markers in the fetal hypothalamus is inversely correlated to maternal body weight and circulating insulin levels. Other circulating factors in the dam that are elevated during an obese pregnancy, such as glucose, free fatty acids or cholesterol, could also play a role [36]. Glucose, which freely crosses the placenta, could therefore cause fetal hyperinsulinaemia. As insulin plays a central role in cell proliferation, fetal hyperinsulinemia, resulting in downregulation of insulin signalling components (as has been shown in adult peripheral tissues), may be an underlying cause of resistance to the proliferative effects of insulin in fetal tissues. Importantly, the reduction in growth of fetal hypothalamic NPCs from an obese pregnancy, grown as neurospheres, remains correlated to maternal body weight even after 8 days in culture*,* suggesting permanent programmed changes in mRNA or protein expression in these cells in addition to any immediate effects of maternal and/or fetal circulating factors during pregnancy. It has long been known that insulin is required for neuronal survival and development, and although it has been shown that offspring of obese mothers develop insulin resistance *in utero* [32], the consequences of insulin resistance for neuronal development were not previously known. Insulin is a component of most cell culture medium due to its potent effects on neuronal growth and survival. It is therefore possible that the reduced growth of neurospheres generated from an obese pregnancy is due to programmed insulin resistance in fetal hypothalamic NPCs that therefore fail to respond to the growth-promoting effects of insulin in culture.

Our findings show that the Notch/Hes signalling pathway is activated in the neonatal hypothalamus in response to maternal obesity. The increased levels of hypothalamic Notch and Hes5, and decreased expression of Ngn2, that we observed in the offspring of obese mothers can at least in part explain the downregulation of hypothalamic NPC proliferation and reduction in mature neuronal markers in these animals. Our results corroborate previous studies that have shown that maternal obesity activates Notch/Hes signalling in NPCs and inhibits neuronal maturation in offspring in other areas of the brain [23,25]. As the Hes family supress the pro-neural transcription factor Mash (*Ascl1*) and neurogenins, we may have expected to observe a concomitant decrease in *Ascl1* expression in response to the increased levels of *Hes5*, although it is possible that the observed decrease in *Ngn2* is sufficient to explain the reduction of neuronal markers. However, this warrants further investigation as disruption of Mash in the hypothalamus causes an imbalance of orexigenic and anorexigenic neurons [44,45] and would therefore also explain our observation of an altered ratio of *Npy* to *Pomc* in the ARC. Furthermore, a reduction of hypothalamic Mash has been reported in another model of maternal obesity in offspring at PND28, accompanied by an increase in NPY-positive neurons [42]. There is therefore a possibility that hypothalamic *Ascl1* mRNA may be decreased at a later stage in the offspring of obese mothers from our model.

There is a strong synergy between Notch and insulin signalling pathways in peripheral tissues, particularly those involved in glucose homeostasis [46]. Furthermore, Notch and insulin signalling interact to control signalling cascades that are fundamental to cell growth [47], although this has not been studied extensively in fetal/neonatal tissues during organ development. Interestingly, increased Notch signalling is associated with insulin resistance in adult adipose tissue and liver [[48], [49], [50]], and inhibition of Notch signalling ameliorates diet-induced insulin resistance [51]. The relationship between Notch and insulin signalling is reciprocal; physiological levels of insulin regulate components of the Notch signalling pathway in adult muscle and liver [52], and a non-canonical insulin-dependent Notch activation pathway promotes proliferation of muscle stem cells [53]. Furthermore, exposure of human pancreatic islets to high glucose has also been shown to regulate genes enriched in the Notch/Hes signalling pathway [54]. As fetal hyperinsulinaemia in the context of an obese pregnancy is thought to be induced by maternal and consequently fetal hyperglycaemia, this provides another route by which a maternal obese environment can influence perinatal Notch signalling and subsequently neuronal development.

Our results show that exposure to maternal obesity alters the ratio of anorexigenic to orexigenic signals in the ARC, as has been reported previously [55,56]. These changes are associated with an inability of the hypothalamus to coordinate energy homeostasis when faced with a metabolic challenge (e.g., a high calorie diet or overnight fast) and precede the increases in adiposity observed in the offspring of obese mothers. Developmental windows in the hypothalamus are defined and precise due to the time restricted expression of transcription factors and circulating hormones [57,58], and therefore the changes we observed in normal hypothalamic developmental processes in the context of an obese pregnancy are likely causative of dysregulation in hypothalamic feeding pathways.

In conclusion, our novel results show that exposure to maternal obesity disrupts key developmental processes and reveal for the first time that this includes hallmarks of insulin resistance in the developing hypothalamus. These changes in the developing hypothalamus are associated with a disrupted neuropeptide profile and feeding behaviour in adult offspring of obese mothers. We propose that fetal hypothalamic insulin resistance may underlie the development of long-term feeding dysregulation in offspring exposed to maternal obesity, and if translatable to humans these findings provide an insight into therapeutic options to limit the inter-generational transmission of obesity risk.
